# Medical and Philosophical Intelligence

**Published:** 1819-11

**Authors:** 


### Medical and Philosophical Intelligence.

WE extract the following history from the Edinburgh Medical Journal (July 1819), as an illustration of the insidious progress of *chronic inflammation of the stomach:* the value of it consists in the salutary advice it should convey to the medical practitioner.

"John Davis, æt. 19, by trade a shoemaker, about three years ago became affected with pain and irritability of the stomach, so that little solid food could be taken without exciting uneasiness and sickness, with violent retching. However, nothing came from the stomach but a viscid mucus, or a watery fluid. From the frequency of these attacks during the last twelve months, he became thin, weakly, and unable to work at his trade. Often, within this time, the epigastric region acquired an excessive hardness and tumefaction. He had great pain and vomiting, which continued until two or three quarts of chocolate-coloured granulated fluid was ejected. After this he remained tolerably easy for ten days or a fortnight, when a recurrence of the same symptoms was experienced.

"On Christmas-day, after attending divine service, he complained, as usual, of his stomach. In the evening intense pain was felt in the epigastrium. He was exceedingly sick; and, with a view to excite vomiting, he drank warm water and chamomile-tea, and also took two active emetics; but these had no effect in evacuating the stomach. Pain, fullness, and extreme tension, increased, and extended to the whole abdomen. Injections *per anum* were administered, and anodynes given, without affording the smallest relief. At two o'clock the next morning he expired.

*Examination.*—The abdomen contained three or four quarts of a dark-coloured fluid. The omentum and intestines were vascular; and, with the peritoneum, exhibited numerous spots of extravasation. Towards the lesser curvature, and near the cardiac orifice, the stomach was mortified, and had given way to a considerable extent. The stomach was also unusually large, its coats thick, and the mucous membrane highly injected with blood. Near the pylorus, it was preternaturally hard, and set round with several whitish tumors, two of which were as large as hazel-nuts. A section of the pyloric orifice showed this outlet very thick, unyielding, and contracted to the size of a common quill."

THE last Number of the Journal above mentioned contains histories of three cases of *poisoning by arsenic,* related by Mr. M'LEOD, which are interesting in several points of view; especially as they show the influence of severe disease of the stomach on the brain; and the results that ensued, in apparently desperate circumstances, from appropriate medical aid. The quantity of the mineral taken appears to have been but small; the patients were three female servants. The violence of the symptoms of inflammation of the stomach in the first instance subsided under the use of blood-letting, castor-oil as a purgative, carbonate of potash, sulphur, and the sulphuret of potash; but, on the third day these symptoms recurred, accompanied with firm closure of the jaws, and convulsions over the whole of the body. On the following day, Mr. M'Leod says, "we found Anne Murray in a state of apoplexy; breathing laborious, with convulsive startings all over her body; her jaws firmly locked; countenance pale and squallid; pulse 90, and feeble; and she was apparently insensible to external objects. We removed her to a cooler apartment, applied a large blister to the pit of the stomach, and another below the chin; took four ounces of blood from one of the jugular veins, and ordered her a strong injection. In about an hour thereafter she could move her jaws; when the physic [purgatives?] and sulphuret of potash were ordered as formerly. At twelve o'clock I sprinkled her face with cold water, which had the effect of rousing her out of the comatous state she was in. She spoke to us at one o'clock, and complained of a violent head-ach, together with heat and pain in her throat. The medicines were continued, and her feet were immersed for some time in warm water. She continued to recover the whole of this day.

"We found Kitty Macintyre speechless, without the power of swallowing, and with her hand carried constantly towards her throat; her face was flushed, her pulse 104 and full, which determined us to take some blood from her arm. Before an ounce came away, she spoke, and said, Thank God! I am relieved. We bled her to the extent of eight ounces; her medicines were repeated; and she continued tolerably easy during the evening, but occasionally complained of head-ach."

The third patient was in a nearly similar state, and was relieved by similar means.

On the following day, (the fifth from the time of taking the poison,) Mr. M'Leod says, "finding them all in a fair way of recovery, I took my leave for that day; but, at nine o'clock in the evening, Anne Murray and Kitty Macintyre were nearly at the same time both seized with violent head-ach, succeeded soon after by high delirium, being insensible of their own situation, unmanageable, and not knowing any of their attendants. They listened to what was said to them, but always gave foolish answers to any questions that were asked. I found them in this state at four o'clock of the morning of Tuesday the 21st; but, as their pulses were very feeble, I did not venture to take any more blood. I gave to each of them a dose of salts, applied a large blister to the nape of their necks, ordered their feet to be immersed in warm water, gave them draughts of gruel with some nitre. But finding, after a trial of five hours of these remedies, that they did not receive the least benefit, (at the same time judging that these secondary symptoms indicated inflammation of the brain,) I determined to try the effects of the affusion of cold water. I accordingly poured some of this several times over the head of the strongest of them, (Kitty Macintyre;) kept at the same time a towel dipped in the cold water to her head. The effects were almost instantaneous: before the application of the water was continued for any length of time, a degree of shivering and gentle hiccough came on, when she recognized all in the room, became calm, and spoke perfectly sensibly. She said she had no pain, but felt a giddiness in her head, and general languor. Shortly after she fell asleep, and wakened quite composed. I tried the same remedy with some confidence in the case of Anne Murray, and the result was exactly similar. Marian Mylis, who was in a separate house, on hearing that the other two were delirious, became so also, though not in so violent a degree. She continued in this state from the preceding day till the affusion of the cold water was used; when the same happy result followed as in the other two cases. This treatment was continued for some days, together with some doses of physic. They gradually recovered strength, without any return of their complaints, from this date; and are now (three months after the accident) in good health."

---

We extract the following interesting case from the American Medical Recorder (vol. i.): it shows, in a very forcible manner, to what a degree derangement of the functions of the brain will extend, from congestion of blood in that organ, without organic lesion. The rapidity of the recovery evinces the correctness of the latter remark.

"I was called, (says Dr. Meigs,) at half-past three o'clock, P.M. on the 18th of June, to see a boy four years old, a fine healthy child as to shape and stature. He had been playing all the morning in a field where his father was mowing. At twelve o'clock he came home, and complained of fatigue and drowsiness; for which his mother sent him into an upper room to sleep. No notice was taken of him till half an hour before my visit, when he was discovered lying on his back quite insensible. His father procured a bleeder, who abstracted about six ounces of blood from his arm, which seemed to have no effect in lessening the violence of the attack.

"When I saw him, he was lying on his back, his countenance pale and death-like, his eyes half-closed, and utterly insensible to common impressions. The corners of his mouth were occupied with a frothy mucus, which was flowing out along with considerable quantities of a brownish fluid. His pulse was yet quite full and strong, though very slow. There was no rigidity of any of the muscles at first, but I at length observed a convulsive clenching of the hand; his abdomen was not swollen, but quite soft and yielding; his breathing performed with some labour, and stertorous.

"I gave him half a tea-spoonful of assafœtida in water, which was swallowed with some difficulty. Sinapisms were applied to the feet; and I laid pounded ice on his head, while cups (with the scarificator) were applied to his temples. He took five grains of calomel, which he could hardly get down, as the function of swallowing was exerted with much difficulty. He started a little when the cupping-lancets struck him, but did not seem to feel the cup.

"In less than an hour my patient was looking about him with much animation, and apparently well of every thing but the cupping scars and sinapisms, of which he complained. He took an ounce of castor-oil, to quicken the operation of the calomel.

"*At* 9, P.M.—He has been sitting up; and I now find him in a placid deep sleep, with a natural pulse and a moist skin."

Dr. Meigs, in his judicious remarks on this case, says, there was no reason to believe that any deleterious substance had been swallowed: he was disposed to attribute it to the influence of the rays of the sun, "during the whole forenoon, on the little fellow's bare head."

---

M. Esquirol lately communicated to the Société Medicale of Paris, an account of a girl who had been hypochondriacal for a long time, and had a disposition to destroy herself. She had been paraplegic for four years, and used crutches: these she threw aside a few hours before her death, walked round the wards of the hospital several times, and then threw herself out of a window. On opening the body, there were found two enormous cysts in the liver,—one in the great lobe, and the other, as it were, placed between the diaphragm and the liver: they contained a large quantity of hydatids. There were several other little cysts also containing them. The largest cyst had contracted adhesions by its superior surface with the diaphragm, which itself adhered to the lungs. The left ovary also contained several hydatids.*

M. Cullellier has found a great number of hydatids in an exostosis of the tibia, which escaped from it by an opening made into the tumor by the actual cautery.

About a hundred and fifty hydatids have been found in the vertebral canal of an epileptic patient.

In a large cyst found in the body of a carrier, who died from accident, there were found numerous hydatids, contained within each other. It was supposed by the anatomist, that the little bubble which rose from a certain point of them, whenever the vessel containing them was moved, was the sucker of the animal.

M. Roux took this occasion to mention that he had recently extirpated a large tumor from the breast of a female, which contained a numerous collection of hydatids, without being accompanied with other appearance of disease of the parts.—*Procés verbaux de la Société de Medecine*, seance du 20 April, 1819.

---

* No notice is taken of the state of the brain and spinal marrow in the report from which the above is extracted. It is probable that hydatids had existed in one or other of them, causing the paralysis; and we may imagine that this affection had been removed by the bursting of them, in consequence of which the compression they had caused may have been removed.—Edit.

The following curious case is taken from the American Medical Recorder: it is related by Dr. Henkel, of Shenandoah county, Virginia.

"On the 31st of May, 1817, a man, about 23 years of age, applied to me to have a tumor extirpated, situated about half an inch above his left eye-brow. It was somewhat larger than a pigeon's egg, and had commenced its growth when he was about nine years old. It was not at all painful, nor was the skin over it discoloured, or otherwise altered. On removing the tumor, and cutting into the sac, I was surprised to find its contents consisted of hair, resembling in every respect those of his eyebrows. After extracting them, I found the inner surface of the cyst covered with hairs, which grew from it. The tumor was no doubt occasioned by the old hairs falling off, and being retained in the cavity."

---

We in the last Number gave an account of the extent of the distribution of vaccine virus from the *National Vaccine Institution;* that from the *London* has been not much inferior. Dr. Walker has distributed, since the last Annual Report, 31,613 charges of matter to 6,223 applicants; and has himself inoculated, within the same period, 2,821 children: the appointed inoculators in the metropolis and its environs, of the same Institution, have inoculated 16,926. Yet, notwithstanding the exertions of those Institutions, small-pox continues to appear. Dr. Walker says, in the same Report, that "almost every day's mails bring urgent applications for vaccine ichor by return of post, from different parts of the United Kingdom, in consequence of the small-pox still existing in the country." It is, however, gratifying to find that the benefits of vaccination have at length extended to almost every part of the world; that the practice is progressively becoming more generally resorted to by every nation; and every year continues to render more firm the reliance that has ever been rationally placed on its prophylactic power.

---

### MONTHLY CATALOGUE OF MEDICAL BOOKS.

### REPORT OF DISEASES.

DURING the early part of the last month, the ordinary diseases of the season prevailed, as diarrhœa, dysentery, intermittent and remittent fevers, especially in children; and hepatic derangement, more or less of an inflammatory character. But the remarkable change that took place about the middle of the month, has brought on the winter epidemics in a very extensive manner, and with unusual severity. Many of those who were suffering diarrhœa and dysentery, have acquired intermittent fever,—an observation made by Morton under analogous circumstances; and many of the more delicate females have become dropsical. Rheumatic affections of the lower extremities have also, in several cases, been complicated with intestinal disorder: these are cases which require much caution and judgment in their treatment. A gentle diarrhœa, either spontaneous or produced by art, has appeared to be the most salutary occurrence, especially in the early stages of the complaint: in the later ones, it has not been productive of much advantage. Inordinate evacuation by the urinary passages has then been most decisive; especially when the urine has been high-coloured and contained albumen.

Inflammation of some part of the respiratory organs, especially of the mucous membrane of the lungs, has been the most common form of fresh attacks of disease. This is more frequently productive of future than immediate fatal consequences: it is the most common cause of phthisis in persons not predisposed to that disease. When the inflammation has totally or nearly subsided, and cough with copious expectoration remains, unalleviated by ipecacuanha, squills, digitalis, and regularly-repeated purgatives every third or fourth day, we have found, in many cases, great benefit derived from balsamic medicines, especially copaiba and turpentine. These are remedies that are too much neglected; but the use of them requires great vigilance in observing their effects.

Croup, and inflammation affecting the trachea more extensively, have been rather prevalent in children, in the northern and eastern districts; and the former affection has been of an intense inflammatory character generally. It is of some importance, in respect to the treatment of this complaint, to ascertain whether or not the voice and respiration are constantly equally shrill and sonorous; as, when this varies much, the child occasionally breathing somewhat freely, especially during *sleep*, which rarely takes place in the more inflammatory species, and if it does happen, the respiration is then more stetorous, it is not advisable to have recourse to such extreme depletion as in the other form of the disease: here, after one venesection, or leeches once applied to the throat, a blister, with active purgatives of calomel and jalap, will soon remove this affection. The constriction of the glottis in these cases, is probably, in a great measure, dependant on functional contraction.

Acute rheumatism, occurring apparently idiopathically, has been common. When we compare the effects of opium, bark, and heating sudorifics, nearly the sole measures employed by most of our forefathers in this disease, with those resulting from bleeding, antimony, cool air and drinks, &c. now generally employed, we know not how the former class of remedies came to be considered beneficial: but many circumstances seem to show that the diseases of the inhabitants of London have of late years assumed a more inflammatory character than they did formerly; though we must not forget, that rheumatism is a term applied in an extremely vague manner, and to affections of the pathology of which we know but very little, that can be considered as precise knowledge.

### METEOROLOGICAL JOURNAL.

By Messrs. WILLIAM HARRIS and Co. 50, Holborn, London.

***From September 20 to October 19, inclusive.***

| Day of Month. | Moon. | Rain guage | THERM. | | | BAROM. | | De Luc's HYGROM. Dry | Damp | | WIND. | | ATMOSPHERIC VARIATION. | | |
|---|---|---|---|---|---|---|---|---|---|---|---|---|---|---|---|
| Sep. | | | | | | | | | | | | | | | |
| 20 | | | 53 | 60 | 46 | 30·33 | 30·38 | | 9 | 8 | NE | NE | Fine | Cloud. | Fine |
| 21 | | | 51 | 61 | 52 | 30·46 | 30·45 | | 9 | 8 | ENE | NE | Fine | | |
| 22 | | | 57 | 60 | 52 | 30·43 | 30·40 | | 8 | 9 | E | ESE | Fine | | |
| 23 | | | 57 | 62 | 54 | 30·23 | 30·07 | – | 9 | 8 | ENE | E | Foggy | Fine | Fine |
| 24 | | | 58 | 63 | 53 | 30·02 | 29·78 | | 11 | 9 | SE | E | Fine | | |
| 25 | | ,32 | 58 | 60 | 53 | 29·63 | 29·58 | | 13 | 12 | E | SW | Rain | | |
| 26 | ☾ | ,13 | 57 | 59 | 54 | 29·56 | 29·71 | | 11 | 15 | SW | SW | Fine | Rain | Cloud. |
| 27 | | ,44 | 59 | 61 | 59 | 29·63 | 29·67 | | 15 | 16 | WSW | SW | Cloud. | Cloud. | Rain |
| 28 | | ,40 | 64 | 65 | 60 | 29·63 | 29·70 | | 17 | 16 | WSW | SSW | Rain | Cloud. | Cloud. |
| 29 | | ,16 | 61 | 62 | 58 | 29·60 | 29·76 | | 17 | 17 | SW | SW | Rain | Rain | Cloud. |
| 30 | | | 63 | 66 | 62 | 29·83 | 29·86 | | 17 | 16 | SW | SW | Cloud. | Fine | Fine |
| Oct. | | | | | | | | | | | | | | | |
| 1 | | | 66 | 70 | 62 | 29·83 | 29·80 | | 15 | 15 | SW | SW | Fine | | |
| 2 | | | 65 | 68 | 61 | 29·70 | 29·83 | | 17 | 15 | SW | WSW | Fine | | |
| 3 | ○ | ,06 | 64 | 64 | 56 | 29·76 | 29·64 | | 15 | 15 | SW | WSW | Cloud. | Rain | Fine |
| 4 | | ,04 | 61 | 62 | 42 | 29·58 | 29·73 | | 14 | 10 | W | N | Cloud. | Sho'ry | Fine |
| 5 | | | 45 | 56 | 45 | 29·94 | 30·16 | | 8 | 8 | N | NW | Fine | Fine | Cloud. |
| 6 | | | 54 | 56 | 54 | 30·13 | 30·00 | | 9 | 11 | W | NW | Cloud. | | |
| 7 | | | 57 | 62 | 58 | 29·90 | 29·95 | | 15 | 15 | W | W | Cloud. | | |
| 8 | | | 60 | 64 | 59 | 30·03 | 30·04 | | 16 | 17 | W | SW | Cloud. | | |
| 9 | | | 61 | 67 | 59 | 29·97 | 29·76 | | 16 | 15 | SSW | SSW | Cloud. | Fine | Cloud. |
| 10 | | | 65 | 68 | 60 | 29·83 | 29·84 | | 15 | 15 | SW | S | Fine | Fine | Cloud. |
| 11 | ☽ | | 63 | 68 | 58 | 29·87 | 29·94 | | 16 | 14 | SE | SW | Fine | | |
| 12 | | | 65 | 69 | 60 | 30·00 | 30·05 | | 15 | 15 | S | SE | Fine | | |
| 13 | | | 65 | 69 | 54 | 30·00 | 30·05 | | 16 | 12 | SW | NW | Cloud. | Cloud. | Fine |
| 14 | | ,05 | 57 | 60 | 51 | 30·10 | 30·18 | | 15 | 10 | W | NNW | Cloud. | Cloud. | Sho'ry |
| 15 | | | 57 | 62 | 45 | 30·30 | 30·34 | | 12 | 11 | NNE | E | Fine | | |
| 16 | | | 53 | 67 | 42 | 30·24 | 30·14 | | 11 | 10 | WNW | NW | Cloud. | Cloud. | |
| 17 | | | 47 | 55 | 41 | 30·14 | 30·20 | | 9 | 9 | N | N | Fine | | |
| 18 | | | 45 | 48 | 39 | 30·20 | 30·20 | | 11 | 9 | NE | N | Fine | | |
| 19 | ● | | 47 | 50 | 51 | 30·10 | 29·94 | | 10 | 12 | NW | SW | Cloud. | Cloud. | Fine |

The quantity of rain fallen in September, is 2 inches and 11-100ths.

